# Intracellular Delivery of Rapamycin From FKBP Elastin-Like Polypeptides Is Consistent With Macropinocytosis

**DOI:** 10.3389/fphar.2018.01184

**Published:** 2018-10-17

**Authors:** Santosh Peddi, Xiaoli Pan, John Andrew MacKay

**Affiliations:** ^1^Department of Pharmacology and Pharmaceutical Sciences, School of Pharmacy of the University of Southern California, Los Angeles, CA, United States; ^2^Department of Pharmaceutical Chemistry, School of Pharmacy of the University of Kansas, Lawrence, KS, United States; ^3^Department of Biomedical Engineering, Viterbi School of Engineering of the University of Southern California, Los Angeles CA, United States; ^4^Department of Ophthalmology, Keck School of Medicine of the University of Southern California, Los Angeles, CA, United States

**Keywords:** confocal microscopy, dynamic light scattering, FK506 binding protein, split luciferase reporter, drug delivery, immunosuppressant, cellular uptake

## Abstract

Rapamycin (Rapa) is a highly potent drug; however, its clinical potential is limited by poor solubility, bioavailability, and cytotoxicity. To improve Rapa delivery, our team has fused the cognate protein receptor for Rapa, FKBP12, to high molecular weight elastin-like polypeptides (ELPs). One construct, FAF, includes an FKBP domain at each termini of an ELP. In a recent report, FAF/Rapa outperformed a family of related carriers with higher tumor accumulation and efficacy. Despite apparent efficacy, an explanation for how FAF carries Rapa into cells has not been elucidated. This manuscript explores the intracellular fate of FAF in MDA-MB-468, a triple negative (ER-/PR-/HER2-) breast cancer line. Based on a lack of displacement by excess unlabeled FAF, no evidence was found for the involvement of a receptor in cell-surface binding. Cellular association showed no dose-dependent saturation at concentrations up to 100 μM, which is consistent with uptake through fluid phase endocytosis. FAF does colocalize with dextran, a marker of fluid phase endocytosis. Upon internalization, both FAF and dextran target low pH intracellular compartments similarly. Despite likely exposure to lysosomal pH and proteolytic activity, intracellular FAF is eliminated from cells with a relatively long half-life of 17.7 and 19.0 h by confocal microscopy and SDS-PAGE respectively. A split luciferase reporter assay demonstrated that FAF delays the cytosolic access of Rapa in comparison to free drug by 30 min. A specific macropinocytosis inhibitor, amiloride, completely inhibits the cytosolic delivery of Rapa from FAF. Each of these results are consistent with macropinocytosis as the mechanism of cellular uptake necessary for the hand-off of Rapa from FKBP-based drug carriers like FAF to endogenous FKBP12 in the cytosol.

## Introduction

Rapamycin (Rapa), also known as Sirolimus is an immunosuppressive agent approved for the prophylaxis of organ rejection during transplantations, especially renal transplants (Dumont and Su, 1995; MacDonald, 2001). Originally discovered in a soil sample on the Easter Island, Rapa is a macrolide produced by the soil bacterium *Streptomyces hygroscopicus* (Kojima et al., 1995). Through inhibition of the mTORC1 complex, it also exhibits potent cytostatic activity and arrested the growth of National Cancer Institute’s NCI60 panel of human tumor cell lines. Although Rapa is not approved for the treatment of cancer, the closely related rapalogue known as Everolimus has been FDA approved for multiple indications in cancer. Rapalogues continue to be evaluated in multiple clinical trials, in combination with other drugs. A major hurdle limiting Rapa’s current clinical utility is its poor drug-like properties. With an undetectable water solubility (Simamora, 2001) and high permeability, it is classified as a Class II drug according to the Biopharmaceutics Classification System (BCS). Rapamune, an oral Rapa formulation exhibits low bioavailability (10–15%) (Trepanier et al., 1998) and poor pharmacokinetics (PK) (Ferron et al., 1997) with a wide variability in inter and intra-patient PK parameters (Augustine et al., 2007; Monchaud and Marquet, 2009). Moreover, treatment is associated with adverse effects including painful oral ulcers (incidence > 30%) (de Oliveira et al., 2011), severe anemia (incidence > 20%), hyperlipidemia, hypercholesteremia, pulmonary and renal toxicities (Marti and Frey, 2005). This limits patient compliance and makes it difficult to maintain patients on Rapa therapy (Gomez-Fernandez et al., 2012), thereby restricting usage to low dosage regimens (Pham et al., 2004).

To improve bioavailability and PK properties, an intravenous route of administration was suggested. Initial attempts to solubilize Rapa to facilitate parenteral injection include the use of organic co-solvents (Sun et al., 2011) like dimethyl acetamide and propylene glycol, and surfactants like polyoxyethylated fatty acids and alcohols (Lorenz et al., 1977; Weiss et al., 1990). However, such co-solvents were found to be toxic (Gelderblom et al., 2001), and further testing in humans was not carried out. While formulation scientists struggled, chemists synthesized analogs of Rapa, called rapalogues to improve the drug’s solubility and other physico-chemical properties. One such rapalogue, Temsirolimus is FDA approved to treat renal cell carcinoma and is administered intravenously. Falsely referred to as ‘water soluble rapalogue,’ temsirolimus for injection contains high proportions of polysorbate 80, polyethylene glycol and alcohol as co-solvents. Polysorbate 80 is associated with infusion related hypersensitivity reactions and can potentially cause anaphylaxis, or even death during administration. Thus, a safe and surfactant-free parenteral formulation of any rapalogue is yet to reach the market. To solve this, we previously employed carrier-assisted delivery and engineered elastin-like polypeptides (ELPs) as drug vehicles for Rapa (Dhandhukia et al., 2017a,b).

**FIGURE 1:**
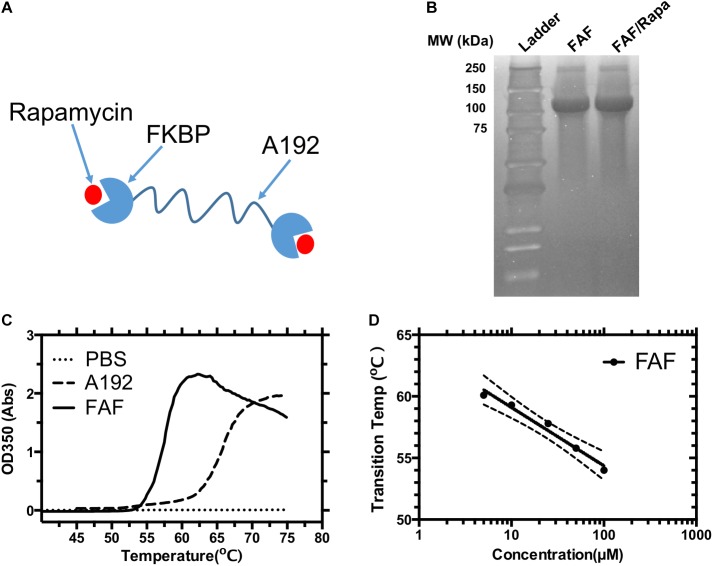
Physicochemical characterization of the FKBP12 elastin-like polypeptide (FAF). **(A)** A schematic diagram of the FAF/Rapa complex (FAF = FKBP-G(VPGAG)_192_-FKBP). **(B)** SDS-PAGE was stained by copper chloride and used to verify the identity and purity of the fusion protein before and after drug loading. **(C)** Optical density vs. temperature profile for 25 μM FAF and control ELP A192. **(D)** The transition temperature (*T*_t_) of FAF was found to be concentration dependent and can be fit to a Log-linear model, *T*_t_ = *b* – *m* [Log_10_ (Concentration)] where *b* represents the *T*_t_ at 1 μM, which is 63.8 ± 2.1°C, *m* represents the *T*_t_ change for a 10-fold change in concentration which is 4.7 ± 1.5°C. These data show that FAF remains soluble at 37°C. *b*, *m* and error bands in the figure represent the Mean ± 95% CI.

Elastin-like polypeptides are protein polymers composed of a pentameric sequence [Val-Pro-Gly-X_aa_-Gly]_n_, where X_aa_ can be any amino acid and n specifies the number of repeats (Urry et al., 1976; Janib et al., 2013). Being genetically encodable, ELP sequences can be manipulated through molecular cloning and synthesized in various heterologous hosts (Despanie et al., 2016), including *E. coli.* As a function of X_aa_ and n, ELPs and their fusion proteins display a characteristic inverse transition temperature (*T_t_*) above which they phase separate into a reversible gel-like coacervate (Despanie et al., 2016). This stimulus responsiveness enables non-chromatographic purification from recombinant expression systems through multiple rounds of inverse phase transition cycling (ITC). To facilitate Rapa delivery, we fused FKBP12, the drug’s cognate receptor to ELP sequences and generated a library of FKBP-ELP drug carriers. While FKBP mediates high affinity drug binding and solubilization, a high molecular weight ELP tag potentially reduces carrier renal clearance and improves plasma half-life. The carrier also sequesters free circulating Rapa, thereby shifting bio-distribution patterns and reducing drug off-target toxicity. After extensive *in vitro* and *in vivo* evaluation of the library, FKBP-[VPGAG]_192_-FKBP (FAF) was identified as the most promising carrier (**Figure 1A**) for Rapa (Dhandhukia et al., 2017a). FAF has a molecular weight of 97 kDa and remains soluble at physiological temperatures (T_t_ > 37°C). It binds to Rapa with high affinity (*K*_d_ = 5 nM), and FAF/Rapa complexes retain Rapa’s cytostatic effect. In a mouse xenograft model of triple negative breast cancer (negative for the estrogen, progesterone, and HER2 receptors), the FAF/Rapa formulation potently inhibited tumor growth and exhibited a better toxicity profile compared to free Rapa control. Although extensively characterized *in vivo*, the mechanisms underlying cellular uptake and drug release from high-affinity FAF/Rapa complexes remain unclear. Using the MDA-MB-468 cell line as the model system, this manuscript aims to better delineate these mechanisms. The generated data can be applied to further optimize the performance of the FAF drug carrier.

## Materials and Methods

### FAF Purification and T_t_ Measurements

Molecular cloning of FAF has previously been described in detail elsewhere. For expression, BLR(DE3) *E. coli* competent cells (Novagen, Madison, WI, United States) were transformed with pET25b(+) vector encoding FAF. Cells were spread on agar plates with 100 μg/mL carbenicillin and incubated overnight at 37°C. A single colony was inoculated to 50 mL autoclaved Terrific Broth (TB) medium (Mo Bio Laboratories, Carlsbad, CA, United States) supplemented with 100 μg/mL carbenicillin and incubated overnight at 37°C in an orbital shaker. Starter culture was then transferred to 1 L TB medium supplemented with 100 μg/mL carbenicillin and allowed to grow for 24 h at 37°C in an orbital shaker. Bacteria were pelleted by centrifuging at 5000 *g* for 15 min, and the pellet was re-suspended in phosphate buffered saline (PBS) (Caisson labs, Smithfield, UT, United States). Bacterial lysis and protein purification by ITC have been previously described elsewhere (Hassouneh et al., 2010). The purified protein was sterile filtered using 200 nm Acrodisc^®^ filters (Pall Corporation, Port Washington, NY, United States). Protein concentration was measured on Nanodrop spectrophotometer using Beer-Lambert’s law, with a correction for light scattering:

(1) $$C=\frac{\left(A_{280}-1.929A_{330}\right)}{\varepsilon l}$$

where C is the solution concentration (M), *A*_280_ and *A*_330_ are absorbance at 280 and 330 nm respectively, *l* is the path length (cm) and ε is the estimated molar extinction coefficient at 280 nm: 20,190 M^−1^cm^−1^ for FAF. The purity and molecular weight of purified FAF were confirmed by SDS-PAGE gel stained with 10% w/v Copper Chloride solution.

To characterize phase transition behavior, varying concentrations of FAF (5–100 μM) were heated from 25 to 75°C at 1°C/min on DU800 spectrophotometer (Beckman Coulter, Brea, CA, United States) and optical density at 350 nm (OD_350_) was measured. T_t_ is defined as the temperature at which OD_350_ (vs) Temperature plot has maximum slope.

### Rhodamine Labeling

NHS-Rhodamine (Thermo Fischer Scientific, Waltham, MA, United States) was dissolved in anhydrous DMSO (Invitrogen, Carlsbad, CA, United States) at 10 mg/ml and frozen as single use aliquots. To 200 μM FAF in PBS, 2x molar excess NHS-Rhodamine was added and incubated at room temperature for 1 h. Zeba desalting columns (Thermo Fischer Scientific, Waltham, MA, United States) were used according to manufacturer’s protocol to remove unreacted free dye and elute Rho-FAF in PBS. Concentrations of rhodamine and FAF were calculated using Nanodrop spectrophotometer as follows:

(2) $$C_{\mathrm{Rho}}=\frac{A_{555}}{\varepsilon l}$$

(3) $$C_{\mathrm{FAF}}=\frac{\left(A_{280}-0.34A_{555}\right)}{\varepsilon l}$$

where *A*_280_ and *A*_555_ are absorbance at 280 nm and 555 nm respectively, *l* is the path length (cm) and ε is the estimated molar extinction coefficient at 280 nm: 20,190 M^−1^cm^−1^ for FAF and 80,000 M^−1^cm^−1^ for rhodamine. Labeling efficiency, *N*, was calculated as:

(4) $$N=\frac{C_{\mathrm{Rho}}}{C_{\mathrm{FAF}}}\times 100$$

The purity of Rhodamine-FAF (Rho-FAF) was evaluated by SDS-PAGE electrophoresis followed by fluorescence imaging on ChemiDoc^TM^ (Bio-Rad, Hercules, CA, United States) imaging system. The concentration of Rho-FAF throughout the manuscript refers to rhodamine concentration unless otherwise specified.

### Dynamic Light Scattering

Hydrodynamic radius (*R*_h_) measurements were obtained from a DynaPro plate reader II (Wyatt Technologies, Santa Barbara, CA, United States) at 37°C. Briefly, 20 μM protein solutions were passed through a 200 nm filter, and 60 μl sample was added in triplicate to a 384-well clear bottom plate (Greiner Bio One, Monroe, NC, United States). The wells were capped with 15 μl mineral oil to prevent evaporation during measurements. Data was analyzed using Dynamics V7 software (Wyatt, Santa Barbara, CA, United States).

### Rapa Encapsulation and Concentration Measurements

To prepare a working formulation of Rapa loaded FAF, a two-phase encapsulation method was employed. 200–400 μM (2 mL) FAF in PBS was equilibrated in a glass vial to 37°C, followed by addition of 3x molar excess Rapa (LC Laboratories, Woburn, MA, United States) in hexane/EtOH mixture (7:3 v/v). After complete evaporation of the organic phase under mild flow of nitrogen, the aqueous suspension was centrifuged at 13,000 *g* at 37°C to pellet unbound Rapa precipitate. The supernatant was subjected to additional rounds of centrifugation until no pellet was observed. FAF/Rapa was added to a 10 kDa MWCO dialysis bag (Thermo Fischer Scientific, Waltham, MA, United States) and dialyzed against PBS (1:750 sample: dialysate) for 12 h to remove free Rapa and residual solvent. An aliquot of encapsulated material was injected onto a C-18 RP-HPLC column (Waters, Milford, MA, United States) and Rapa was quantified at 280 nm using a calibrated standard curve.

### Cell Culture

MDA-MB-468 cell line (HTB-132, ATCC, Manassas, VA, United States) was cultured in Dulbecco’s modified Eagle’s medium (DMEM)/F-12 medium (DFL21, Caisson labs) supplemented with 10% fetal bovine serum (FBS, Corning, NY, United States) in a humidified incubator with 5% CO_2_ at 37°C.

### Cold Competition Binding Assay and Live Cell Imaging

3 × 10^5^ MDA-MB-468 cells were seeded on a 35 mm glass bottom dish (MatTek Corporation, Ashland, MA, United States) and allowed to attach overnight. After 24 h, culture medium was replaced with 750 μL fresh medium supplemented with 25 mM HEPES. The dish was placed on ice and pre-chilled Rho-FAF was added to a final concentration of 20 μM. After a 2-h incubation, a 10-fold excess of unlabeled FAF or an equal volume of cold PBS was added and incubated for another 2 h. Cells were washed three times with PBS and 1 mL live cell imaging solution (Life Technologies, Carlsbad, CA, United States) was added to the dish with two drops of NucBlu^TM^ reagent (Life Technologies, Carlsbad, CA, United States). Images were captured using LSM800 confocal microscope (Carl Zeiss Microscopy, Thornwood, NY, United States) mounted on a vibration-free table with a Plan-Apochromat 63x oil objective. Integrated fluorescence intensities were measured by drawing regions of interest (ROI) on ImageJ software (NIH, Bethesda, MD, United States).

### Concentration-Dependence of Cellular Association

MDA-MB-468 cells were seeded in triplicate on a black bottom 96-well plate (Greiner Bio One, Monroe, NC, United States) at a density of 10,000 cells/well and allowed to attach overnight. Next, culture medium was replaced with 100 μL fresh medium containing 1–100 μM Rho-FAF. After 16 h incubation at 37°C and 5% CO_2_, medium was aspirated and cells were washed three times with PBS. 100 μL live cell imaging solution was added to each well and total fluorescence intensity was measured using a Synergy H1 plate reader (BioTek, Winooski, VT, United States).

### Cellular Uptake and Co-localization Analysis

3 × 10^5^ MDA-MB-468 cells were seeded in a 35 mm glass bottom dish and allowed to attach overnight. The next morning, culture medium was replaced with 1 mL fresh medium containing either 30 μM Rho-FAF, 20 μM Fluorescein-dextran (70 kDa MW, Life Technologies, Carlsbad, CA, United States), 20 μM Rhodamine B-dextran (70 kDa MW, Life Technologies, Carlsbad, CA, United States), or both 30 μM Rho-FAF and 20 μM Fluorescein-dextran (FL-dextran). After an 8-h treatment, medium was aspirated and cells were washed three times with PBS. 1 mL live cell imaging solution was added to each dish with two drops of NucBlu^TM^ reagent. When applicable, 1 μL LysoTracker^TM^ Green DND-26 (LTG, Life Technologies, Carlsbad, CA, United States) was added. Images were captured as described above. For co-localization analysis, Mander’s Co-localization Coefficient (MCC) was calculated for both red and green channel using ZEN2009 software (Carl Ziess Microscopy, Thornwood, NY, United States) using following equations

(5) $$M_{\mathrm{Red}}=\frac{\sum_{i}R_{\mathrm{i,coloc}}}{\sum_{i}R_{i}}M_{\mathrm{Green}}=\frac{\sum_{i}G_{\mathrm{i,coloc}}}{\sum_{i}G_{i}}$$

### Kinetics of Cellular Uptake and Degradation

Cells were seeded in 35 mm dishes as previously described and treated with 30 μM Rho-FAF or 30 μM Rho-FAF/Rapa for 1, 4, 8, 16, and 24 h at 37°C. At each time point, cells were washed, imaged, and analyzed as described. To assess cellular degradation following pulsed incubation, cells treated with 30 μM Rho-FAF or 30 μM Rho-FAF/Rapa for 16 h at 37°C. After that, culture medium was replaced with fresh medium and cells were imaged at 0, 1, 8, 24, 48, and 72 h as described above. After imaging, cells were washed with PBS and lysed using 120 μL RIPA buffer (Thermo Fisher Scientific, Waltham, MA, United States) containing 1x protease/phosphatase inhibitor cocktail (Cell Signaling Technology, Danvers, MA, United States). Total protein concentration in each cell lysate was determined by BCA assay (Thermo Fisher Scientific, Waltham, MA, United States) following manufacturer’s protocol and 22.5 μg total protein was loaded in each well of a 4–20% Tris-Glycine gel. Following SDS-PAGE, the gel was imaged on ChemiDoc^TM^ imaging system.

### Split Luciferase Assay

30,000 cells/well were seeded in triplicate in a white opaque flat bottom 96-well plate (Greiner Bio One, Monroe, NC, United States) and allowed to attach overnight. The next morning, the medium was aspirated and 90 μL/well Opti-MEM medium (Thermo Fisher Scientific, Waltham, MA, United States) was added. Each well was transfected with 50 ng FKBP-SmBit and 50 ng FRB-LgBit (Promega, Madison, WI, United States) using 0.3 μL/well Lipofectamine 3000 (Thermo Fisher Scientific, Waltham, MA, United States) transfection reagent according to manufacturer’s protocol. One-day post-transfection, cells were treated with 1 mM amiloride (Sigma-Aldrich, St. Louis, MO, United States) for 30 min. Following the above treatment, 25 μL/well Nano-Glo^®^ luciferase substrate (Promega, Madison, WI, United States) was added and the plate was equilibrated to room temperature. Cells were treated with either 30 nM Rapa/DMSO or 30 nM FAF/Rapa and immediately placed in a plate reader to measure luminescence with 1 s integration time. Measurements were taken in kinetic mode for 2 h at 30-s intervals.

## Results

### Physicochemical Characterization of FAF

FAF was successfully purified from *E. coli* with yields ranging between 80 and 100 mg/L bacterial culture, as previously reported (Dhandhukia et al., 2017a). Three rounds of heating/cooling were sufficient to produce > 95% pure protein, as visualized by copper stained SDS-PAGE gel (**Figure 1B**). Molecular weight on SDS-PAGE matched the expected molecular weight of 97 kDa. To test if the process of Rapa encapsulation results in any obvious changes in molecular weight or purity of the drug carrier, FAF/Rapa complexes were also resolved on SDS-PAGE gel. No loss of integrity was observed as FAF/Rapa displayed equivalent molecular weight and purity as FAF.

To confirm that FAF remains soluble at all temperatures evaluated herein, the optical density of FAF solutions was measured as a function of temperature at various concentrations (**Figure 1C**). The lower critical solution temperature (LCST) phase separation of ELPs is well-studied (Urry, 1988, 1992). As temperature increases, ELPs lose the water network solvating the polypeptide backbone through entropy driven processes thereby increasing their hydrophobicity. This is accompanied by a gradual conformational change toward more ordered secondary structures, predominantly type-2 β spirals. Together, they result in ELP assembly and coacervation over a narrow temperature range, usually 1–2°C, which can be detected as a sharp increase in solution turbidity. As reported previously, FAF exhibits concentration dependent phase transition properties with the T_t_ dropping slightly from 60 to 54°C as concentration increases from 5 to 100 μM (**Figure 1D**). Hence, at experimental temperature of 37°C, FAF remains soluble across all the concentrations relevant to this manuscript.

### Rhodamine Labeling and Rapa Encapsulation

**FIGURE 2:**
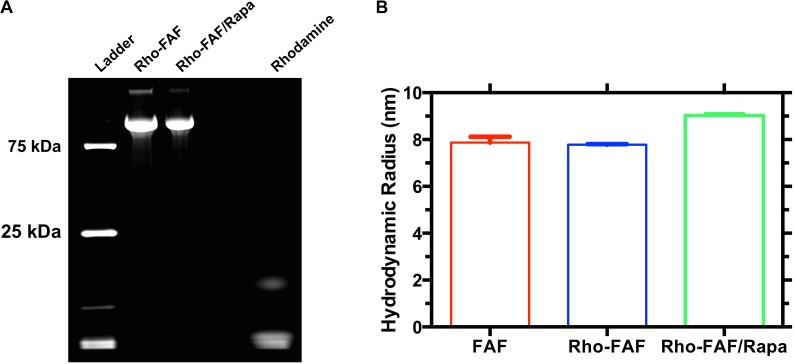
Rhodamine-labeled FAF with and without Rapamycin is pure and stable. FAF was labeled with NHS-Rhodamine and then used to encapsulate rapamycin. **(A)** SDS-PAGE imaged by fluorescence indicates successful labeling and complete removal of unreacted dye. **(B)** Dynamic light scattering was used to estimate the hydrodynamic radii of FAF, Rho-FAF, and Rho-FAF/Rapa. While there was an ∼1 nm increase in the radius of Rho-FAF/Rapa compared to Rho-FAF, no relevant particle populations of drug or protein aggregates appeared. Aggregation would have yielded hydrodynamic radii more than an order of magnitude larger than FAF alone. Hence, neither rhodamine labeling nor drug encapsulation induced aggregation or physical instability (*n* = 3, Mean ± SD).

To enable tracking by fluorescence microscopy, we labeled FAF with rhodamine fluorophore. NHS activated rhodamine tagged lysine side chains in FAF through an amide linkage. Incubation with twofold excess dye yielded an efficiency of 160%, indicating an average of 1.6 rhodamine molecules in each FAF molecule. Labeling strategies generally impact substrate properties, thereby rendering good quality control practices essential. Since small amounts of unreacted dye in purified Rho-FAF can cause experimental artifacts, we tested our purification technique using SDS-PAGE followed by fluorescence imaging (**Figure 2A**). Rho-FAF was completely free of unreacted dye and any other impurities. To ensure labeling did not induce any physical instability or aggregation, hydrodynamic radii (*R_h_*) were measured using DLS. At 37°C, FAF and Rho-FAF displayed a 7.8 nm radius with no significant differences in size (**Figure 2B**). A working formulation of Rapa bound Rho-FAF (Rho-FAF/Rapa) was prepared by two-phase encapsulation as described earlier. After removal of unbound Rapa, HPLC was used to quantify solution concentrations of Rho-FAF and Rapa. Encapsulation ratio (ER), defined as the ratio of Rapa to Rho-FAF concentration was found to be 1.8 ± 0.2 (*n* = 3, Mean ± SD). Since ER is close to 2, we can conclude that both the FKBPs in Rho-FAF can bind and solubilize Rapa. This further suggests the structure of FAF remains properly folded after rhodamine labeling.

### Cellular Uptake of Rho-FAF Is Receptor Independent

**FIGURE 3:**
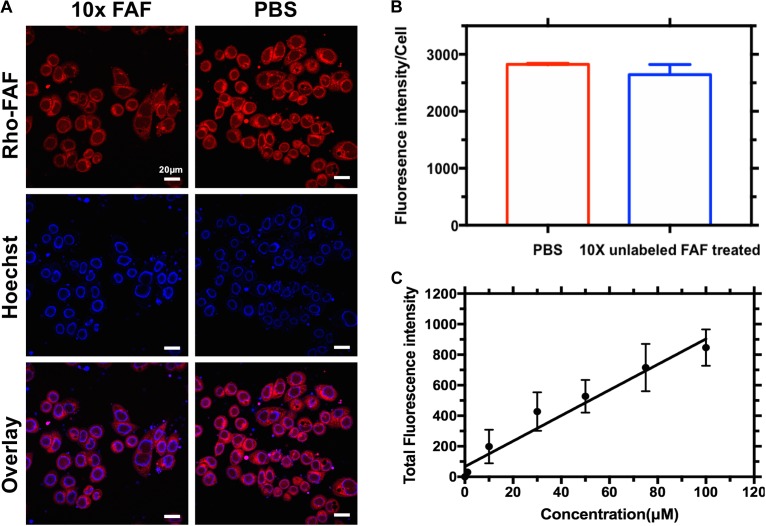
Cellular association of Rho-FAF is non-saturable. **(A,B)** MDA-MB-468 cells were pre-incubated in complete media with Rho-FAF (20 μM) on ice for 2 h, supplemented with either unlabeled FAF (125 μM) or an equal volume of PBS, and incubated again for 2-h on ice. Cells were washed and live cell imaging was then performed using laser scanning confocal microscopy. Red: Rho-FAF; Blue: Hoechst 33342; Scale bar: 20 μm. The integrated fluorescence intensity per cell was determined using ImageJ (*n* = 32, Mean ± SD). Excess unlabeled FAF was unable to displace Rho-FAF. **(C)** MDA-MB-468 cells in complete media were treated with increasing concentrations of Rho-FAF at 37°C for 16 h, washed, and the total fluorescence intensity was quantified using a plate reader. The cell-associated fluorescence was linearly related to the incubation concentration, *r*^2^ = 0.9. The apparent non-saturation of cellular association is consistent with macropinocytosis (*n* = 4–6, Mean ± SD).

When MDA-MB-468 cells were incubated with Rho-FAF at 4°C, washed and imaged at room temperature using live cell imaging, a bright ring staining pattern on the cell surface was observed (**Figure 3A**). This was accompanied by low-level internalization and cytoplasmic distribution of rhodamine signal, which may have occurred during short exposures to room temperature during imaging. At 4°C, all endocytic pathways are arrested with minimal disruption of affinity driven binding, thereby allowing detection of binding events at the plasma membrane. When competed with 10-fold molar excess unlabeled FAF, there was no significant decrease in cell surface rhodamine staining and the integrated fluorescence intensity per cell remained constant (**Figure 3B**). This suggests absence of a specific receptor that can bind to and mediate FAF internalization. Concentration-dependent cellular uptake of Rho-FAF was next determined. A defining feature of receptor-mediated endocytosis is saturability at high ligand concentrations. When cells were incubated with 1–100 μM Rho-FAF for a constant time period, the total amount of internalized protein increased linearly with concentration (*R*^2^ = 0.9) with no evidence of saturation (**Figure 3C**). This further supports a receptor-independent cellular uptake mechanism for FAF.

### Cellular Uptake by Macropinocytosis and Translocation to Low pH Compartments

**FIGURE 4:**
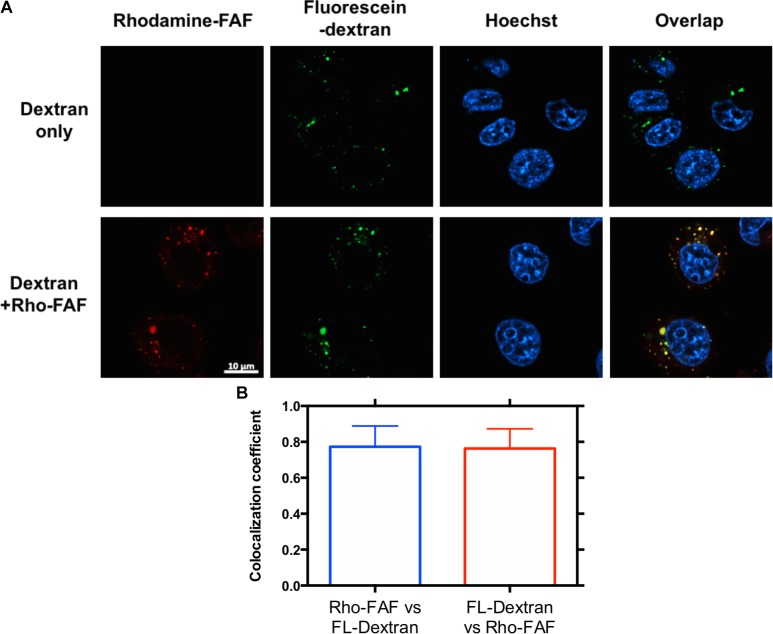
Rho-FAF and FL-dextran display equivalent distribution after cellular uptake. **(A)** MDA-MB-468 cells were incubated with FL-dextran at 37°C in complete media, to mark macropinocytotic uptake for 8 h, washed with PBS, and imaged by laser scanning confocal microscopy. When cells were co-incubated with Rho-FAF (30 μM) and FL-dextran (20 μM), images showed high co-localization between red and green channels, which is consistent with macropinocytosis as the mechanism for Rho-FAF internalization. Red: Rho-FAF; Green: FL-dextran; Blue: Hoechst 33342. **(B)** Mander’s correlation coefficient (MCC) obtained in individual cells were averaged to 0.77 ± 0.12, 0.76 ± 0.11 for Rho-FAF, FL-dextran respectively (*n* = 16, Mean ± SD).

**FIGURE 5:**
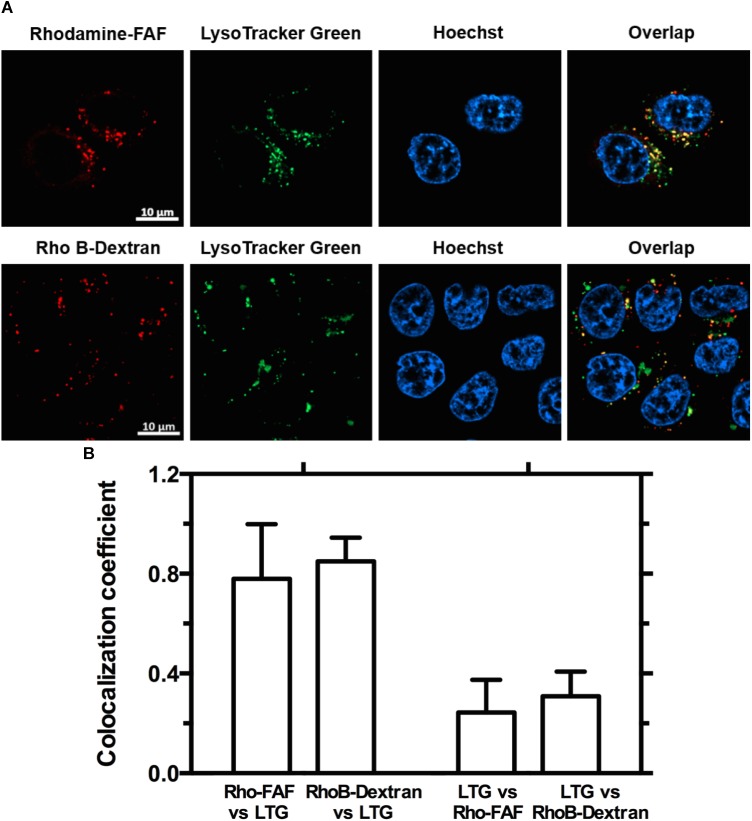
Both Rho-FAF and Rho-dextran localize to low pH compartments upon cellular uptake. **(A)** MDA-MB-468 cells were either incubated in complete media with Rho-FAF or RhoB-dextran for 8 h followed by addition of Lyso-Tracker Green (LTG) before laser scanning confocal imaging. Images show high overlap of the red channel within pixels positive in the green channel, which indicates that both Rho-FAF and Rho B-dextran accumulate in low pH compartments. While a high degree of Rho-FAF and Rho B-dextran colocalized with LTG, a significant amount of low pH compartments do not contain Rho-FAF or RhoB-dextran. Both observations are consistent with macropinocytic vesicles. Red: Rho-FAF or RhoB-dextran; Green: LTG; Blue: Hoechst 33342. **(B)** Co-localization analysis in individual cells revealed a shared pattern wherein a large fraction of intracellular FAF and dextran were associated with a small fraction of acidic organelles (*n* = 12, Mean ± SD).

Macropinocytosis is an ATP dependent, non-specific uptake of extracellular fluid and solutes through membrane protrusions that collapse onto and fuse with the cell membrane thereby generating large endocytic vesicles called macropinosomes (Lim and Gleeson, 2011). FITC-dextran is a widely used marker for macropinocytosis. When co-incubated with Rho-FAF and imaged using confocal microscopy, equivalent cellular distribution of both Rho-FAF and FITC-dextran was observed (**Figure 4A**). Consequently, superimposed images had abundant yellow pixels, consistent with colocalization. To quantify colocalization, the MCC for Rho-FAF (red channel) and FITC-dextran (green channel) were estimated as 0.77 ± 0.12 and 0.76 ± 0.11 respectively, which reflect a high degree of cellular co-localization (**Figure 4B**). No leakage of FITC fluorescence into the rhodamine channel was detected when cells incubated with FITC-dextran only were imaged. These results strongly support macropinocytosis as the mechanism for FAF internalization. To study the intracellular fate of FAF, Lyso Tracker Green (LTG) was used to label low pH organelles including lysosomes (**Figure 5A**). The MCC for Rho-FAF and LTG were 0.78 ± 0.22 and 0.24 ± 0.13 respectively (**Figure 5B**), suggesting a large proportion of cellular Rho-FAF is associated with a small subset of acidic organelles. These organelles may play a role in FAF degradation or drug release from FAF/Rapa complexes. Similar cellular distribution and co-localization coefficients were obtained with the positive control Rho-dextran (**Figures 5A,B**), which is known to translocate to lysosomes after macropinocytosis.

### Time-Dependent Cellular Uptake and Cellular Degradation

**FIGURE 6:**
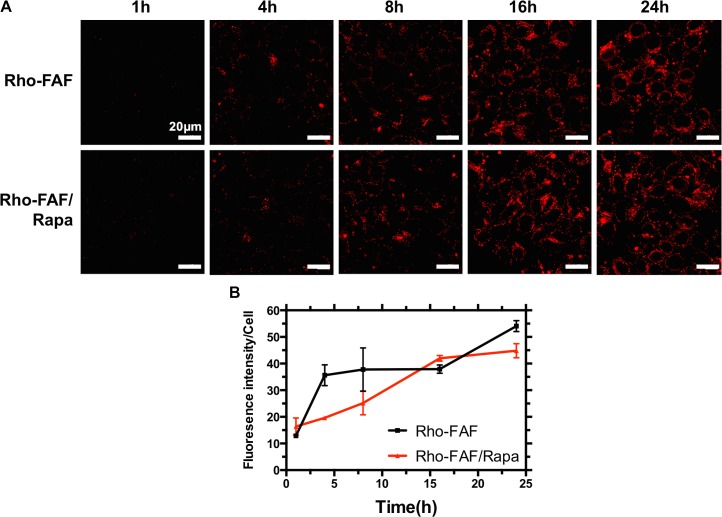
The kinetics of cellular association of Rho-FAF is minimally affected by addition of Rapa. **(A)** MDA-MB-468 cells were incubated with Rho-FAF (30 μM) in complete media at 37°C, washed at the indicated time points, and imaged using laser scanning confocal microscopy. Increased fluorescence signal was observed with longer incubation time points; however, the addition of Rapa had no dramatic effect on the kinetics of cellular uptake. **(B)** At least three images of each time point were analyzed for fluorescence intensity and cell number. Fluorescence intensity/cell against time profile showed time-dependent uptake (*n* = 3–7, Mean ± SD).

**FIGURE 7:**
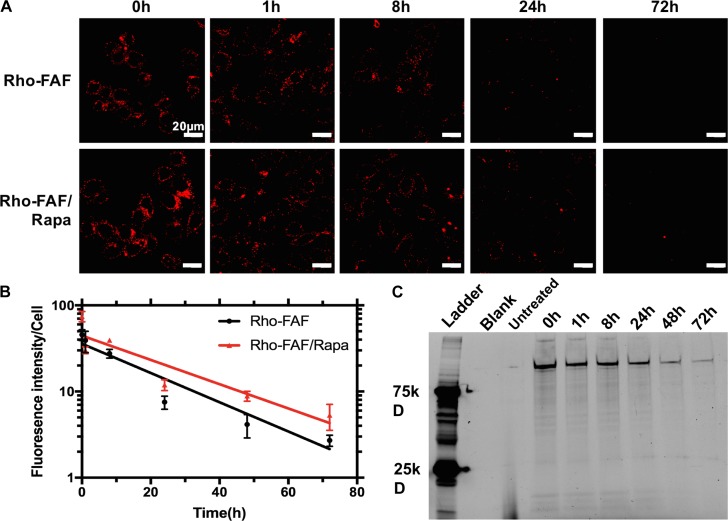
Loss of cellular Rho-FAF is minimally affected by addition of Rapa. **(A)** MDA-MB-468 cells were pulse incubated with either Rho-FAF or Rho-FAF/Rapa for 16 h at 37°C. After multiple washes, cells were incubated in complete media for the indicated time points and imaged using laser scanning confocal microscopy. A decrease in fluorescence was observed with time consistent with the degradation, dilution, or cellular export of FAF. **(B)** Fluorescence intensity per cell was measured using image analysis and plotted against time (*n* = 3–7, Mean ± SD). Degradation followed one-phase decay with a half-life of 17.7 h (15.6–20.4 h, 95% CI) and 21.3 h (17.7–26.5 h, 95% CI) for Rho-FAF and Rho-FAF/Rapa respectively. **(C)** Cell-associated fluorescence was recovered from cells and observed by SDS-PAGE. The band for Rho-FAF diminished with time, along with appearance of multiple low molecular weight species. The estimated half-life for Rho-FAF using densitometry was 19.0 h (15.4–21.6 h, 95% CI), which is in close agreement with confocal images.

To evaluate the kinetics of uptake, cells incubated with Rho-FAF or Rho-FAF/Rapa for 1 to 24 h were imaged (**Figure 6A**). Increasing incubation time consistently resulted in higher uptake in both the groups indicating faster internalization than degradation throughout the timescale measured (**Figure 6B**). To study degradation kinetics, cells were pulsed with Rho-FAF or Rho-FAF/Rapa and imaged up to 3 days after withdrawal of treatment. Confocal images clearly show decreasing cell associated fluorescence with time (**Figure 7A**). The plot of fluorescence intensity/cell against time followed a one-phase decay and the cellular half-lives of Rho-FAF and Rho-FAF/Rapa were estimated to be 18 and 21 h respectively (**Figure 7B**). After imaging, cells were lysed and the lysate was resolved using SDS-PAGE followed by fluorescence imaging. Consistent with microscopy data, Rho-FAF band intensity diminished with time with concomitant appearance of some low molecular weight bands (**Figure 7C**), presumably intermediate degradation products. Lysate of untreated cells served as a negative control and did not show any detectable bands.

### Split Luciferase Assay

**FIGURE 8:**
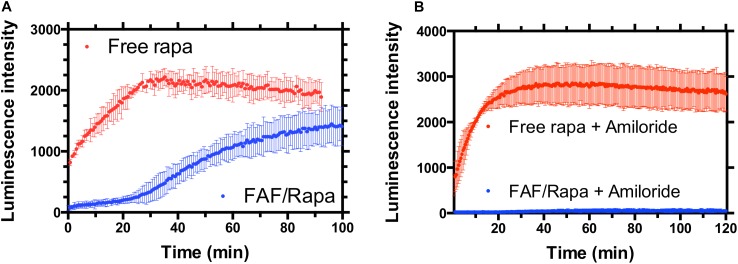
FAF delays the access of Rapa to the cytosol in a manner consistent with macropinocytosis. **(A)** MDA-MB-468 cells were transfected with a split luciferase reporter that enables the specific detection of Rapa within the cytosol. When incubated with cells, free drug resulted in rapid luciferase activity consistent with diffusion across the plasma membrane as the mechanism of cellular entry. In contrast, FAF/Rapa produced luminescence only after 30-min, a period of time consistent with cellular uptake. **(B)** Addition of the macropinocytosis inhibitor amiloride completely blocks cytosolic detection of Rapa from FAF/Rapa. Free Rapa luminescence kinetics were unaffected by amiloride (Mean ± 95% CI, *n* = 6).

To study the uptake and release mechanisms of FAF/Rapa, cells were co-transfected with plasmids encoding FKBP-SmBit and FRB-LgBit. These fusion proteins together function as a sensor for cytoplasmic Rapa. Upon access to the cytoplasm, free Rapa induces dimerization of FKBP and FRB domains, thereby constituting a functional luciferase enzyme. In the presence of furimazine, luciferase catalyzes its conversion to furimamide with concomitant emission of luminescence. Treatment of cells with free Rapa resulted in a rapid luciferase activity with Rapa diffusing across the plasma membrane even prior to obtaining the first measurement. On the other hand, cells treated with FAF/Rapa produced luminescence with a very clear lag time of approximately 30 min, which suggests a different, slower mechanism of drug release to the cytoplasm compared to free Rapa (**Figure 8A**). To better understand the underlying mechanisms, we repeated the assay using cells pre-treated with various inhibitors. Amiloride, a selective inhibitor of macropinocytosis (West et al., 1989; Koivusalo et al., 2010) completely arrested Rapa release from FAF with no significant effect on uptake of free Rapa (**Figure 8B**). Considered in the context of the other experiments in this manuscript, these results provide three conclusions (i) FAF/Rapa complexes retain binding sufficient to prevent an early burst in cytosolic Rapa as seen with the free drug control; (ii) FAF traffics to low pH compartments, which may include lysosomes; and (iii) macropinocytosis of FAF/Rapa complexes is required for Rapa release to the cytoplasm.

## Discussion

This manuscript describes mechanisms of uptake and drug release from FAF, a previously studied carrier for the challenging drug Rapa. Mammalian target of Rapamycin complex 1 (mTORC1) is a nutrient sensing multi-protein complex (Hara et al., 2002) that strongly promotes cell growth and proliferation. Rapa, through inhibition of mTORC1 kinase activity, arrests cell cycle progression from G1 to S phase thereby exerting a cytostatic effect. Deregulation of multiple elements of mTORC1 signaling have been reported in many types of cancers (Guertin and Sabatini, 2005), including melanoma, breast cancer, and renal cell carcinoma, which renders the pathway an attractive therapeutic target. Unfortunately, the clinical outcomes with mTOR inhibitors have been modest, primarily because of their cytostatic and not cytotoxic effects, partial inhibition of mTOR activities, and existence of several feedback loops involved in cell survival responses (Shah et al., 2004; Shi et al., 2005; O’Reilly et al., 2006). Nonetheless, mTOR inhibitors in combination with a wide variety of chemotherapeutics are an active area of research and a number of clinical trials are underway. An additional factor hampering their clinical efficacy is poor drug-like properties, a problem that may possibly be improved using FAF/Rapa formulation.

A variety of internalization mechanisms have been reported for ELP biomaterials. The uptake of Cell penetrating peptide-ELP (CPP-ELP) fusion proteins (Bidwell and Raucher, 2010) was driven by caveolae-independent mechanisms. ATP depletion and inhibition of clathrin-mediated endocytosis had no significant effect on CPP-ELP internalization. On the other hand, the uptake of GFP-K72 in A549 cells (Pesce, 2015) was found to be primarily through caveolae-mediated endocytosis with no contribution from other known mechanisms. Similarly, based on the size of endocytic vesicles, ELP[Val_5_Ala_3_Gly_2_]_150_ was proposed to be internalized by macropinocytosis (Raucher and Chilkoti, 2001) with enhanced uptake upon coacervation. Taken together, it is difficult to predict or compare uptake mechanisms across ELPs or ELP fusion proteins since they differ in their components and properties like charge, size, hydrophobicity, etc. In the present study, FAF co-localized with dextran, a marker for macropinocytosis and its cellular association was not saturable across a range of achievable concentrations (**Figure 3**). Moreover, amiloride, a specific macropinocytosis inhibitor completely blocked the cellular entry of Rapa upon FAF/Rapa treatment (**Figure 8**). These results together support macropinocytosis as the mechanism for FAF/Rapa uptake. Although no evidence for a specific receptor for FAF was observed, the presence of bright surface staining in the cold binding experiment (**Figure 3A**) suggests FAF may weakly and non-specifically bind to molecules of the plasma membrane, presumably lipids. Termed ‘adsorptive endocytosis’ (Lloyd and Williams, 1984), such behavior has been reported with ELPs (Raucher and Chilkoti, 2001) and may be advantageous as high surface concentrations can drive their uptake.

A carrier system that utilizes macropinocytosis for drug delivery poses both advantages and disadvantages. Efficiency of receptor-mediated endocytosis is dependent on expression levels of the internalizing receptor, rendering the process heterogeneous across cell types. On the contrary, macropinocytosis is a generally accepted hallmark of cancer with multiple cancer types exploiting the process in various ways to establish and maintain their oncogenic phenotype (White, 2013; Ha et al., 2016). This suggests a broad-spectrum cytostatic activity for FAF/Rapa against mTOR driven cancers. On the downside, a carrier system utilizing macropinocytosis for drug delivery can exhibit off target side effects since most normal cells, especially macrophages and dendritic cells depend on either ligand induced or constitutive macropinocytosis for normal physiological function (Norbury, 2006; Liu and Roche, 2015). This may result in cellular entry of FAF/Rapa to non-cancerous cells, thereby causing off-target effects.

The ability to undergo biodegradation make ELPs excellent materials compared to some polymeric, inorganic, and metallic nanoparticles. We and other groups previously reported enzymatic degradation of ELPs by trypsin (Nettles et al., 2010), elastase and collagenase (Shah et al., 2012). In a physiological system, these proteases could act intra or extracellularly, or both. In cells, Rho-FAF disappeared with a half-life of 18 h, during which time it significantly colocalizes with low pH compartments, including lysosomes (**Figures 4**, **5**). Interestingly, both elastase (Menninger et al., 1981) and collagenase (Everts et al., 1995) have been identified in the lysosomes, which suggests they might be involved in FAF degradation. While plausible, additional experiments are needed for definitive proof. It must be noted that the drop in cell-associated fluorescence in the biodegradation experiment is a combined effect of biodegradation, cellular recycling/export, and dilution due to cell division. In the Rho-FAF group, there was a 15-fold drop in fluorescence after 72 h relative to the start of experiment. With the doubling time of MDA-MB-468 cells being about 48 h and the experiment lasting for 72 h, only a maximum of threefold drop in fluorescence can be explained by cell division alone. Rho-FAF/Rapa on the other hand may be less prone to dilution due to cell-division, because of Rapa’s cytostatic effect (Dhandhukia et al., 2017a). The biodegradation experiment can be strengthened by complementing SDS-PAGE with a western blot using anti-FKBP and anti-ELP antibodies. Fluorophores, especially during long incubation times are prone to oxidation and other chemical reactions that can cause photobleaching. In such cases, western blots may be reliable alternative to quantify FAF. Nevertheless, with a cellular half-life of 21 h, the persistence of Rho-FAF/Rapa greatly exceeds the approximately 0.5 h time-scale required for intracellular delivery of Rapa (**Figure 8**).

When added to cells, Rapa rapidly diffused across the membrane resulting in spontaneous luciferase activity (**Figure 8**). This is in accordance with a previous report showing near quantitative accumulation in human smooth muscle cells within few minutes of Rapa treatment (Zhu et al., 2009). On the contrary, FAF/Rapa required 30 min before measurable luciferase activity was achieved suggesting mechanisms other than trans-membrane diffusion mediate drug release. If FAF/Rapa complexes caused burst release extracellularly, FAF/Rapa would have produced a similarly rapid luciferase profile as free Rapa. More importantly, amiloride completely blocked cytoplasmic entry of Rapa from FAF/Rapa, thus establishing the requirement for internalization in general, by macropinocytosis in particular. This is in agreement with microscopy experiments that showed Rho-FAF highly co-localized with the macropinocytosis marker dextran (**Figures 4**, **5**). Although proven to selectively inhibit macropinocytosis without any effects on coat dependent endocytosis (West et al., 1989; Dowrick et al., 1993; Koivusalo et al., 2010), amiloride may inhibit clathrin (Meier et al., 2002) and lipid raft mediated internalization (Wadia et al., 2004). Hence, the use of multiple inhibitors can strengthen the proposed role of macropinocytosis. We used dynasore to evaluate the dynamin dependence of FAF/Rapa uptake. In the split luciferase assay, pretreatment of cells with dynasore completely suppressed luciferase signals triggered by both free Rapa and FAF/Rapa (**Supplementary Figure S1B**). Since dynamin inhibition cannot arrest diffusion of free Rapa across the cell membrane, the observed effect is likely an assay interference caused by dynasore by unknown mechanisms.

Similar to the luciferase assay, microscopy experiments do not support a requirement for carrier biodegradation in drug release. The half-life for Rho-FAF/Rapa degradation was found to be 21 h, which is much longer than the time scale for triggering luciferase activity. To test the possible role of endosomal/lysosomal pH in drug release through rapid disruption of FAF/Rapa binding, the luciferase assay was performed using cells pretreated with lysosomal acidification inhibitors chloroquine and ammonium chloride. This did not result in any measurable change in carrier performance (**Supplementary Figure S1A**). Although these findings point toward low pH of acidic organelles not being necessary in Rapa release from FAF/Rapa, such conclusions can be made only after confirming lysosomal basification under the treatment conditions employed.

Another possible mechanism that hasn’t been explored is a release-independent pathway wherein FAF/Rapa directly binds to mTOR and inhibits its kinase activity. When free Rapa is added to cells, it diffuses across the membrane, binds to endogenous FKBPs with high affinity and FKBP/Rapa complexes target mTOR. Since FAF/Rapa structurally resembles naturally occurring FKBP/Rapa, the possibility exists that it may directly bind to and inhibit mTOR. Such a process would not require drug exchange from FAF/Rapa to cellular FKBPs for mTOR inhibition. This can be envisioned as FAF/Rapa may escape endosomes and target cytoplasmic mTORC1. It is well-known that active mTORC1 attaches to the surface of lysosomes (Sancak et al., 2008, 2010), and the microscopy experiments suggest Rho-FAF/Rapa accumulates in the lysosomes (**Figure 5**). Such close proximity due to translocation to the same cellular compartment might conceivably facilitate FAF/Rapa-mTOR interaction across disruptions in the lysosomal membrane. This would require FAF/Rapa to withstand affinity in the luminal milieu with a pH < 5 and about 100 potent hydrolases. To verify a direct FAF/Rapa-mTOR interaction, anti-mTOR co-immunoprecipitation (co-IP) was performed after treating cells with FAF/Rapa. Unfortunately, previously reported cell lysis conditions for anti-mTOR co-IP preserved the mTORC1 complex but disrupted FKBP/Rapa-mTOR interaction (**Supplementary Figure S2**), thereby rendering the experiment inconclusive. As an alternative to co-IP, we used immunofluorescence to detect mTOR Rho-FAF/Rapa co-localization. While microscopy is limited by resolution in identifying direct molecular interactions, a strong co-localization would be supportive of binding. Upon incubating cells with Rho-FAF/Rapa for 1, 8, and 16 h, co-localization was observed (**Supplementary Figure S3**) in a small population of cells (<20%) but the average co-localization coefficient was a mere 0.13. Hence, while it cannot be ruled out, a direct FAF/Rapa-mTOR interaction is unlikely.

## Conclusion

This report characterizes the cell uptake and drug release mechanisms from FAF, an engineered carrier for the challenging drug Rapa. Unlike conventional drug carriers, the FAF/Rapa formulation does not require toxic co-solvents or surfactants and instead utilizes Rapa’s cognate receptor FKBP12 for high-affinity binding-mediated drug delivery. In MDA-MB-468 cells, FAF was internalized by macropinocytosis and accumulated in low pH organelles, which include lysosomes. Cellular uptake was dose and time-dependent but was not saturable. FAF/Rapa is a biodegradable carrier with a cellular half-life of 21 h; however, it appears to deliver its Rapa cargo to the cytosol within a time scale of 0.5 h. Drug release from FAF/Rapa was found to be sensitive to amiloride, a macropinocytic inhibitor. While future studies must be performed to determine the relative importance of other mechanisms of endocytosis, the findings in this manuscript are consistent with macropinocytosis. These results may now be applied to further enhance the *in vivo* performance of drug carriers related to FAF.

## Author Contributions

JM conceived the study. XP and SP collected, analyzed, and interpreted data. SP drafted the manuscript.

## Conflict of Interest Statement

SP and JM are inventors on a patent describing subcutaneous delivery of small molecules using protein-polymer fusions related to this work. The remaining author declares that the research was conducted in the absence of any commercial or financial relationships that could be construed as a potential conflict of interest.

## References

[B1] Augustine J. J. Bodziak K. A. Hricik D. E. (2007). Use of sirolimus in solid organ transplantation. *Drugs* 67 369–391. 10.2165/00003495-200767030-00004 17335296

[B2] Bidwell G. L. III Raucher D. (2010). Cell penetrating elastin-like polypeptides for therapeutic peptide delivery. *Adv. Drug Deliv. Rev.* 62 1486–1496. 10.1016/j.addr.2010.05.003 20478348 PMC2964383

[B3] de Oliveira M. A. Martins F. M. Wang Q. Sonis S. Demetri G. George S. (2011). Clinical presentation and management of mTOR inhibitor-associated stomatitis. *Oral Oncol.* 47 998–1003. 10.1016/j.oraloncology.2011.08.009 21890398

[B4] Despanie J. Dhandhukia J. P. Hamm-Alvarez S. F. MacKay J. A. (2016). Elastin-like polypeptides: therapeutic applications for an emerging class of nanomedicines. *J. Control. Release* 240 93–108. 10.1016/j.jconrel.2015.11.010 26578439 PMC5767577

[B5] Dhandhukia J. P. Li Z. Peddi S. Kakan S. Mehta A. Tyrpak D. (2017a). Berunda polypeptides: multi-headed fusion proteins promote subcutaneous administration of rapamycin to breast cancer In Vivo. *Theranostics* 7:3856. 10.7150/thno.19981 29109782 PMC5667409

[B6] Dhandhukia J. P. Shi P. Peddi S. Li Z. Aluri S. Ju Y. (2017b). Bifunctional elastin-like polypeptide nanoparticles bind rapamycin and integrins and suppress tumor growth in Vivo. *Bioconjug. Chem.* 28 2715–2728. 10.1021/acs.bioconjchem.7b00469 28937754 PMC5779105

[B7] Dowrick P. Kenworthy P. McCann B. Warn R. (1993). Circular ruffle formation and closure lead to macropinocytosis in hepatocyte growth factor/scatter factor-treated cells. *Eur. J. Cell Biol.* 61 44–53. 8223707

[B8] Dumont F. J. Su Q. (1995). Mechanism of action of the immunosuppressant rapamycin. *Life Sci.* 58 373–395. 10.1016/0024-3205(95)02233-3 8594303

[B9] Everts V. Korper W. Niehof A. Jansen I. Beertsen W. (1995). Type VI collagen is phagocytosed by fibroblasts and digested in the lysosomal apparatus: involvement of collagenase, serine proteinases and lysosomal enzymes. *Matrix Biol.* 14 665–676. 10.1016/S0945-053X(05)80030-7 9057816

[B10] Ferron G. M. Mishina E. V. Zimmerman J. J. Jusko W. J. (1997). Population pharmacokinetics of sirolimus in kidney transplant patients. *Clin. Pharmacol. Therap.* 61 416–428. 10.1016/S0009-9236(97)90192-2 9129559

[B11] Gelderblom H. Verweij J. Nooter K. Sparreboom A. (2001). Cremophor EL: the drawbacks and advantages of vehicle selection for drug formulation. *Eur. J. Cancer* 37 1590–1598. 10.1016/S0959-8049(01)00171-X 11527683

[B12] Gomez-Fernandez C. Garden B. C. Wu S. Feldman D. R. Lacouture M. E. (2012). The risk of skin rash and stomatitis with the mammalian target of rapamycin inhibitor temsirolimus: a systematic review of the literature and meta-analysis. *Eur. J. Cancer* 48 340–346. 10.1016/j.ejca.2011.11.028 22206873

[B13] Guertin D. A. Sabatini D. M. (2005). An expanding role for mTOR in cancer. *Trends Mol. Med.* 11 353–361. 10.1016/j.molmed.2005.06.007 16002336

[B14] Ha K. D. Bidlingmaier S. M. Liu B. (2016). Macropinocytosis exploitation by cancers and cancer therapeutics. *Front. Physiol.* 7:381. 10.3389/fphys.2016.00381 27672367 PMC5018483

[B15] Hara K. Maruki Y. Long X. Yoshino K.-I. Oshiro N. Hidayat S. (2002). Raptor, a binding partner of target of rapamycin (TOR), mediates TOR action. *Cell* 110 177–189. 10.1016/S0092-8674(02)00833-4 12150926

[B16] Hassouneh W. Christensen T. Chilkoti A. (2010). Elastin-like polypeptides as a purification tag for recombinant proteins. *Curr. Protoc. Prot. Sci.* 61:CHAPTER: Unit–6.11. 10.1002/0471140864.ps0611s61 20814933 PMC3076942

[B17] Janib S. M. Liu S. Park R. Pastuszka M. Shi P. Moses A. (2013). Kinetic quantification of protein polymer nanoparticles using non-invasive imaging. *Integr. Biol.* 5 183–194. 10.1039/c2ib20169k 23093022 PMC3762326

[B18] Koivusalo M. Welch C. Hayashi H. Scott C. C. Kim M. Alexander T. (2010). Amiloride inhibits macropinocytosis by lowering submembranous pH and preventing Rac1 and Cdc42 signaling. *J. Cell Biol.* 188 547–563. 10.1083/jcb.200908086 20156964 PMC2828922

[B19] Kojima I. Cheng Y. Mohan V. Demain A. (1995). Carbon source nutrition of rapamycin biosynthesis in *Streptomyces hygroscopicus*. *J. Ind. Microbiol.* 14 436–439. 10.1007/BF01573954 7662284

[B20] Lim J. P. Gleeson P. A. (2011). Macropinocytosis: an endocytic pathway for internalising large gulps. *Immunol. Cell Biol.* 89:836. 10.1038/icb.2011.20 21423264

[B21] Liu Z. Roche P. A. (2015). Macropinocytosis in phagocytes: regulation of MHC class-II-restricted antigen presentation in dendritic cells. *Front. Physiol.* 6:1. 10.3389/fphys.2015.00001 25688210 PMC4311620

[B22] Lloyd J. B. Williams K. E. (1984). *Non-Specific Adsorptive Pinocytosis.* London: Portland Press Limited. 10.1042/bst0120527 6734911

[B23] Lorenz W. Reimann H.-J. Schmal A. Dormann P. Schwarz B. Neugebauer E. (1977). Histamine release in dogs by Cremophor EL^®^ and its derivatives: oxethylated oleic acid is the most effective constituent. *Agents Actions* 7 63–67. 10.1007/BF01964882 67784

[B24] MacDonald A. S. (2001). A worldwide, phase III, randomized, controlled, safety and efficacy study of a sirolimus/cyclosporine regimen for prevention of acute rejection in recipients of primary mismatched renal allografts. *Transplantation* 71 271–280. 10.1097/00007890-200101270-00019 11213073

[B25] Marti H.-P. Frey F. J. (2005). Nephrotoxicity of rapamycin: an emerging problem in clinical medicine. *Nephrol. Dial. Transpl.* 20 13–15. 10.1093/ndt/gfh639 15632347

[B26] Meier O. Boucke K. Hammer S. V. Keller S. Stidwill R. P. Hemmi S. (2002). Adenovirus triggers macropinocytosis and endosomal leakage together with its clathrin-mediated uptake. *J. Cell Biol.* 158 1119–1131. 10.1083/jcb.200112067 12221069 PMC2173207

[B27] Menninger H. Burkhardt H. Röske W. Ehlebracht W. Hering B. Gurr E. (1981). Lysosomal elastase: effect on mechanical and biochemical properties of normal cartilage, inhibition by polysulfonated glycosaminoglycan, and binding to chondrocytes. *Rheumatol. Int.* 1 73–81. 10.1007/BF00541157 6287562

[B28] Monchaud C. Marquet P. (2009). Pharmacokinetic optimization of immunosuppressive therapy in thoracic transplantation: part II. *Clin. Pharmacokinet.* 48 489–516. 10.2165/11317240-000000000-00000 19705921

[B29] Nettles D. L. Chilkoti A. Setton L. A. (2010). Applications of elastin-like polypeptides in tissue engineering. *Adv. Drug Deliv. Rev.* 62 1479–1485. 10.1016/j.addr.2010.04.002 20385185 PMC2935943

[B30] Norbury C. C. (2006). Drinking a lot is good for dendritic cells. *Immunology* 117 443–451. 10.1111/j.1365-2567.2006.02335.x 16556257 PMC1782244

[B31] O’Reilly K. E. Rojo F. She Q.-B. Solit D. Mills G. B. Smith D. (2006). mTOR inhibition induces upstream receptor tyrosine kinase signaling and activates Akt. *Cancer Res.* 66 1500–1508. 10.1158/0008-5472.CAN-05-2925 16452206 PMC3193604

[B32] Pesce D. (2015). *Thermotropic Liquid Crystals from Engineered Polypeptides.* Groningen: University of Groningen.

[B33] Pham P.-T. T. Pham P.-C. T. Danovitch G. M. Ross D. J. Gritsch H. A. Kendrick E. A. (2004). Sirolimus-associated pulmonary toxicity. *Transplantation* 77 1215–1220. 10.1097/01.TP.0000118413.92211.B6 15114088

[B34] Raucher D. Chilkoti A. (2001). Enhanced uptake of a thermally responsive polypeptide by tumor cells in response to its hyperthermia-mediated phase transition. *Cancer Res.* 61 7163–7170. 11585750

[B35] Sancak Y. Bar-Peled L. Zoncu R. Markhard A. L. Nada S. Sabatini D. M. (2010). Ragulator-Rag complex targets mTORC1 to the lysosomal surface and is necessary for its activation by amino acids. *Cell* 141 290–303. 10.1016/j.cell.2010.02.024 20381137 PMC3024592

[B36] Sancak Y. Peterson T. R. Shaul Y. D. Lindquist R. A. Thoreen C. C. Bar-Peled L. (2008). The Rag GTPases bind raptor and mediate amino acid signaling to mTORC1. *Science* 320 1496–1501. 10.1126/science.1157535 18497260 PMC2475333

[B37] Shah M. Hsueh P. Y. Sun G. Chang H. Y. Janib S. M. MacKay J. A. (2012). Biodegradation of elastin-like polypeptide nanoparticles. *Prot. Sci.* 21 743–750. 10.1002/pro.2063 22434766 PMC3403411

[B38] Shah O. J. Wang Z. Hunter T. (2004). Inappropriate activation of the TSC/Rheb/mTOR/S6K cassette induces IRS1/2 depletion, insulin resistance, and cell survival deficiencies. *Curr. Biol.* 14 1650–1656. 10.1016/j.cub.2004.08.026 15380067

[B39] Shi Y. Yan H. Frost P. Gera J. Lichtenstein A. (2005). Mammalian target of rapamycin inhibitors activate the AKT kinase in multiple myeloma cells by up-regulating the insulin-like growth factor receptor/insulin receptor substrate-1/phosphatidylinositol 3-kinase cascade. *Mol. Cancer Ther.* 4 1533–1540. 10.1158/1535-7163.MCT-05-0068 16227402

[B40] Simamora P. (2001). Solubilization of rapamycin. *Int. J. Pharm.* 213 25–29. 10.1016/S0378-5173(00)00617-7 11165091

[B41] Sun M. Si L. Zhai X. Fan Z. Ma Y. Zhang R. (2011). The influence of co-solvents on the stability and bioavailability of rapamycin formulated in self-microemulsifying drug delivery systems. *Drug Dev. Ind. Pharm.* 37 986–994. 10.3109/03639045.2011.553618 21417621

[B42] Trepanier D. J. Gallant H. Legatt D. F. Yatscoff R. W. (1998). Rapamycin: distribution, pharmacokinetics and therapeutic range investigations: an update. *Clin. Biochem.* 31 345–351. 10.1016/S0009-9120(98)00048-4 9721433

[B43] Urry D. Okamoto K. Harris R. Hendrix C. Long M. (1976). Synthetic, crosslinked polypentapeptide of tropoelastin: an anisotropic, fibrillar elastomer. *Biochemistry* 15 4083–4089. 10.1021/bi00663a026 963023

[B44] Urry D. W. (1988). Entropic elastic processes in protein mechanisms. I. Elastic structure due to an inverse temperature transition and elasticity due to internal chain dynamics. *J. Prot. Chem.* 7 1–34. 10.1007/BF01025411 3076447

[B45] Urry D. W. (1992). Free energy transduction in polypeptides and proteins based on inverse temperature transitions. *Prog. Biophys. Mol. Biol.* 57 23–57. 10.1016/0079-6107(92)90003-O 1549698

[B46] Wadia J. S. Stan R. V. Dowdy S. F. (2004). Transducible TAT-HA fusogenic peptide enhances escape of TAT-fusion proteins after lipid raft macropinocytosis. *Nat. Med.* 10:310. 10.1038/nm996 14770178

[B47] Weiss R. B. Donehower R. Wiernik P. Ohnuma T. Gralla R. Trump D. (1990). Hypersensitivity reactions from taxol. *J. Clin. Oncol.* 8 1263–1268. 10.1200/JCO.1990.8.7.1263 1972736

[B48] West M. A. Bretscher M. S. Watts C. (1989). Distinct endocytotic pathways in epidermal growth factor-stimulated human carcinoma A431 cells. *J. Cell Biol.* 109 2731–2739. 10.1083/jcb.109.6.2731 2556406 PMC2115909

[B49] White E. (2013). Exploiting the bad eating habits of Ras-driven cancers. *Genes Dev.* 27 2065–2071. 10.1101/gad.228122.113 24115766 PMC3850091

[B50] Zhu W. Masaki T. Cheung A. K. Kern S. E. (2009). In-vitro release of rapamycin from a thermosensitive polymer for the inhibition of vascular smooth muscle cell proliferation. *J. Bioequival. Bioavail.* 1 3–12. 20190878 10.4172/jbb.1000002 PMC2829311

