# Supplementary material for: Intracellular Delivery of Rapamycin From FKBP Elastin-Like Polypeptides Is Consistent With Macropinocytosis
Source: Front Pharmacol. 2018 Oct 17;9:1184. doi: 10.3389/fphar.2018.01184 (PMC6199897; doi:10.3389/fphar.2018.01184)
Supplement: Supplementary file 1 [file Data_Sheet_1.PDF]

# Supplementary data for

# Intracellular Delivery of Rapamycin from FKBP Elastin-like Polypeptides is Consistent with Macropinocytosis

Santosh Peddi[1#], Xiaoli Pan[2#], J. Andrew MacKay[1,3,4 *]

1. Department of Pharmacology and Pharmaceutical Sciences, University of Southern California, Los Angeles, CA, United States
2. Department of Pharmaceutical Chemistry, University of Kansas, Lawrence, KS, United States
3. Department of Biomedical Engineering, University of Southern California, Los Angeles, CA, United States
4. Department of Ophthalmology, University of Southern California, Los Angeles, CA, United States

# These authors contributed equally to this work

Corresponding author
J. Andrew MacKay, PhD.
jamackay@usc.edu
Department of Pharmacology and Pharmaceutical Sciences
School of Pharmacy
University of Southern California
1985 Zonal Avenue
Los Angeles 90033-9121

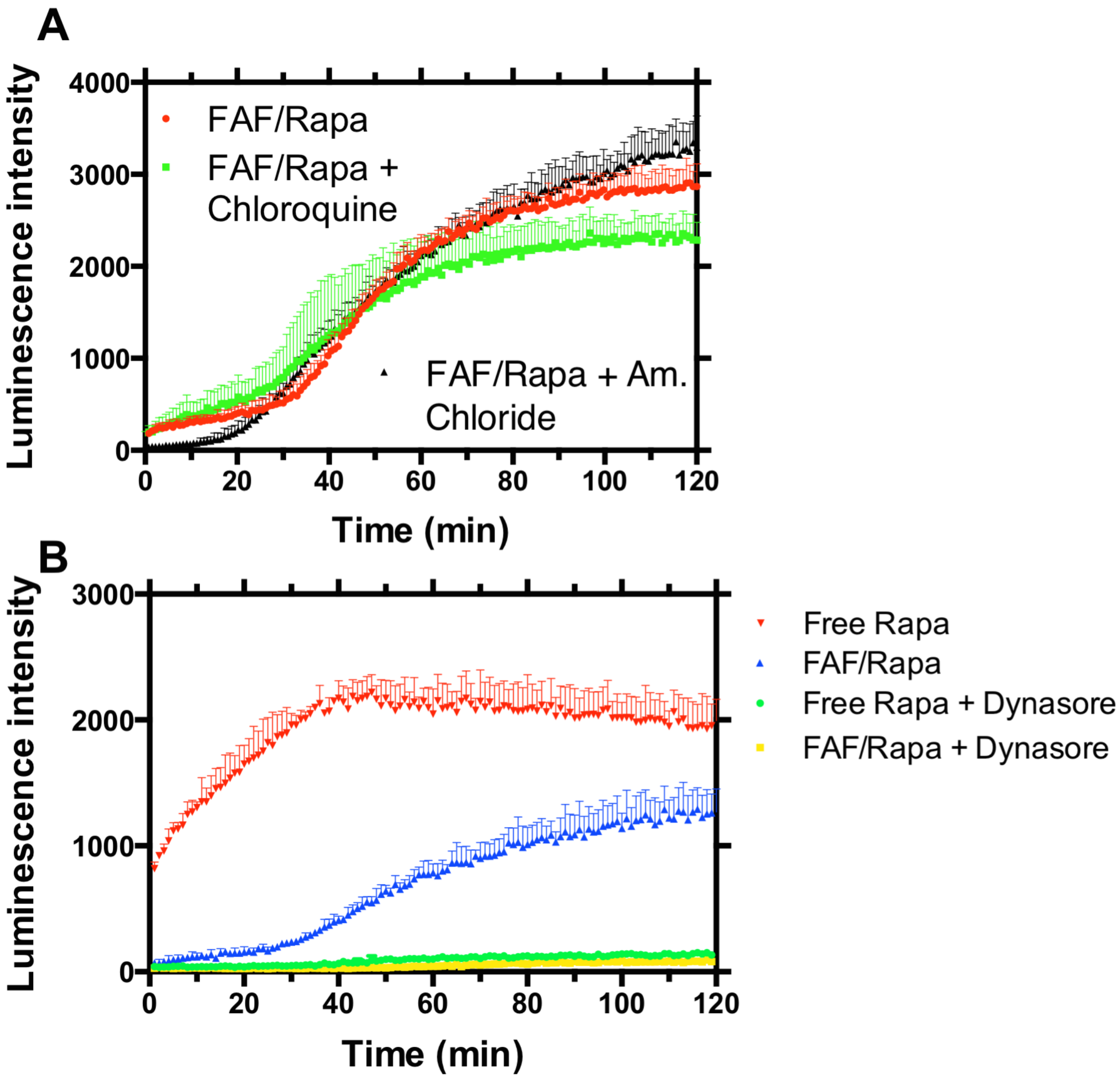


**Figure S1**: A) Inhibition of lysosomal acidification does not inhibit Rapa release from FAF/Rapa. MDA-MB-468 cells were transfected with a split luciferase reporter that enables the specific detection of Rapa within the cytosol as in **Fig. 8**. When incubated with cells, FAF/Rapa (30 nM Rapa) resulted in delayed luciferase activity consistent with endocytosis across the plasma membrane. Two inhibitors of lysosomal acidification were added to determine if they affect the kinetics of Rapa delivery. Neither Chloroquine (80 μM) nor Ammonium Chloride (25 mM) prevented the appearance of a luciferase signal. (Mean ± SD, n=3) B) Dynasore mediated inhibition of dynamic dependent endocytosis resulted in suppression of luciferase activity for both free rapa and FAF/Rapa. Since dynamin inhibition cannot arrest diffusion of free Rapa across the cell membrane, the observed effect is likely an assay interference caused by dynasore by unknown mechanisms.

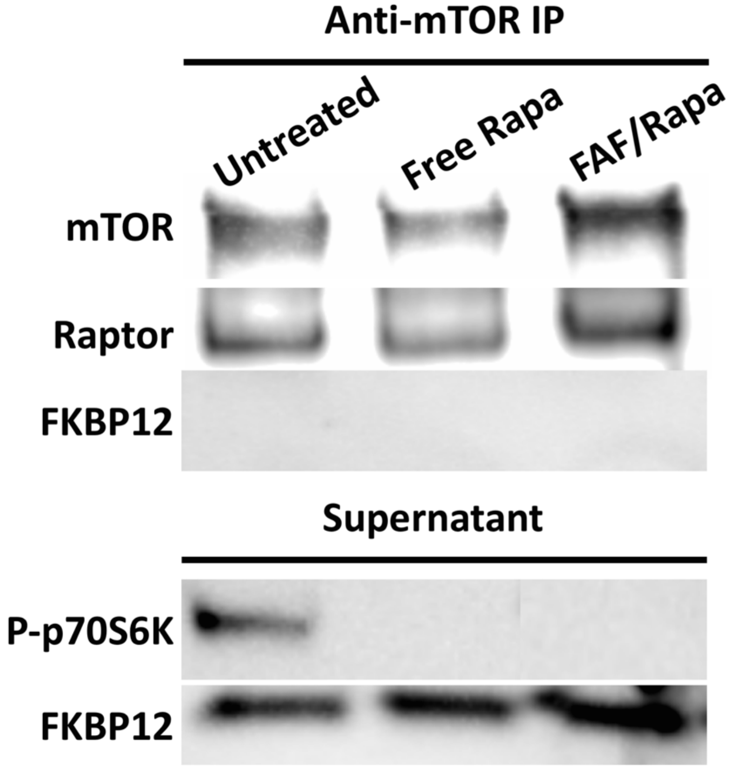


**Figure S2**: Anti-mTOR immunoprecipitation of cell lysates treated with Rapa and FAF/Rapa fail to detect FKBP12-Rapa-mTOR interaction. Cells were treated with Rapa (100 nM) or FAF/Rapa (100 nM) for 30 minutes and lysed in CHAPS lysis buffer. Following anti-mTOR IP, the immunoprecipitated and the supernatant fraction were resolved by SDS-PAGE and the indicated markers were detected by western blotting. Lysis conditions preserved mTORC1 complex as indicated by presence of Raptor in the IP product. On the other hand, the IP conditions interfered with FKBP12/Rapa-mTOR binding resulting in detection of FKBP12 in the supernatant rather than IP fraction. Suppression of p70S6K phosphorylation is indicative of successful Rapa delivery to the cytoplasm and subsequent inhibition of mTOR kinase activity.

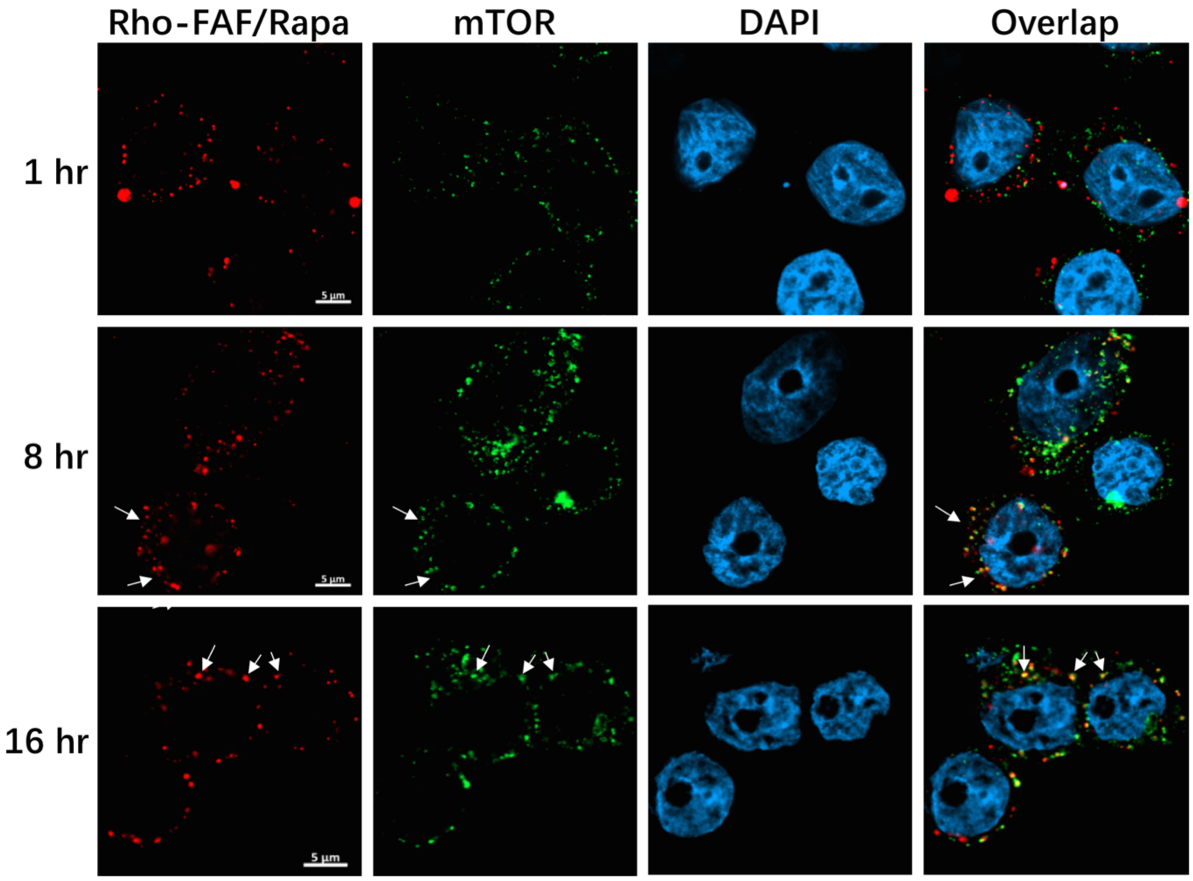


**Figure S3**: Co-localization of FAF/Rapa and mTOR using immunofluorescence. Secondary immunofluorescence was used to assess the intracellular distribution over time for mTOR in relation to Rho-FAF. When MDA-MB-468 cells were treated with FAF/Rapa for 1, 8 and 16 hr, a few areas of co-localization were seen as indicated by arrows. While some cells after 8 hours show evidence of co-localization, the average co-localization co-efficient across many cells was only 0.13. This suggests an unlikely direct interaction between FAF/Rapa and mTOR on time-scales relevant to intracellular delivery of Rapa (**Figure 8**).
